# Influence of the Physiological Pacifier on the Development of Malocclusions in Children: A Scoping Review

**DOI:** 10.3390/children11111353

**Published:** 2024-11-07

**Authors:** Carolina Caleza-Jiménez, Inés Rodríguez Romero, David Ribas-Perez, María Biedma-Perea

**Affiliations:** Department of Pediatric Dentistry, Faculty of Dentistry, University of Seville, C/Avicena S/N, 41009 Seville, Spain; ccaleza@us.es (C.C.-J.); inerodrom@alum.us.es (I.R.R.); mbiedma1@us.es (M.B.-P.)

**Keywords:** pacifier, malocclusion, anterior open bite, overjet, posterior crossbite

## Abstract

Background: As a result of the dental alterations pacifiers can cause, several designs have been described, differing in the shape and size of the teat. The aim of this review was to compare the influence of the physiological pacifier on the development of malocclusions in children with other types of pacifier. The research question was: does the use of physiological pacifiers cause less dentomaxillary alterations than other designs? Methods: A scoping review was carried out according to the Preferred Reporting Items for Systematic Reviews and Meta-Analyses (PRISMA) using PubMed, Embase, and Scopus. The ROBINS-I risk of bias tool was used for the methodology assessment of the included studies. Results: Of the 122 articles identified in the initial search, 5 articles met all the inclusion criteria. In all of them, in general, the use of the pacifier caused malocclusions such as an anterior open bite, a posterior crossbite, an increased overjet, and an involvement of the overbite. Children who began using the physiological pacifier very early, between 0 and 3 months, were less likely to develop finger sucking/thumb sucking compared to children who started after 3 months. In the prevalence of open anterior bite and overjet, there was a significant difference between the use of conventional pacifiers and anatomical pacifiers compared to the use of physiological pacifiers. Conclusions: the physiological pacifier can cause fewer oral alterations and could be the best option as a pacifier, however, more well-designed and high-quality randomised clinical trials are required.

## 1. Introduction

The importance of oral habits is due to their ability to modify the position of the teeth and the pattern of skeletal growth. This occurs due to the alteration of the balance between the forces of the perioral musculature and the tongue during the development of craniofacial structures [1].

As these habits are established from an early age, their elimination is more complicated, increasing the severity of the problems they cause [2]. Establishing harmful oral habits has a multifactorial origin, its presence being related to psychological and environmental aspects [1].

Within the classification of harmful oral habits, there are functional alterations, such as immature swallowing or oral breathing. On the other hand, there are sucking habits, which, in turn, are divided into nutritious and non-nutritive. In this first category we find breastfeeding and the use of a bottle, while among the non-nutritive we find the use of a pacifier, digital sucking, and lip sucking [1].

Sucking is an innate reflex of the individual and is normal in the neonatal period, which stops at 4 months. From this moment on, it goes from being innate to being an acquired act. The baby’s sucking habit is essential when it comes to satisfying nutritional and psychological needs, since it is its means of exchange with the outside world. When nutrition is not satisfactory due to a lack of sucking, the baby looks for other substitute methods to satisfy this instinct [2]. The type of non-nutritive sucking habit or comforting habit and its characteristics, such as prolongation over time, have a different impact on the oral cavity. These should disappear as the child grows, whether driven by himself or by his parents or caregivers [3,4].

The pacifier is used as a method to calm the child and is culturally widespread in many countries, also promoting the well-being of parents and avoiding digital sucking. This fact makes it the most prevalent non-nutritive sucking habit [5]. Its use has been defended because it has been related in different investigations to a preventive effect on sudden infant death syndrome, however, it is not sufficiently evidenced [6,7]. However, pacifier use should not begin before 15 days after birth, so breastfeeding stabilizes [8]. It allows anteroposterior movements of the jaw, but its persistence over time causes alterations such as anterior open bite, lingual interposition, increased overjet, tendency to class II, and, in some cases, crossbites, due to the formation of a high and narrow palate [5,9].

**Figure 1:**
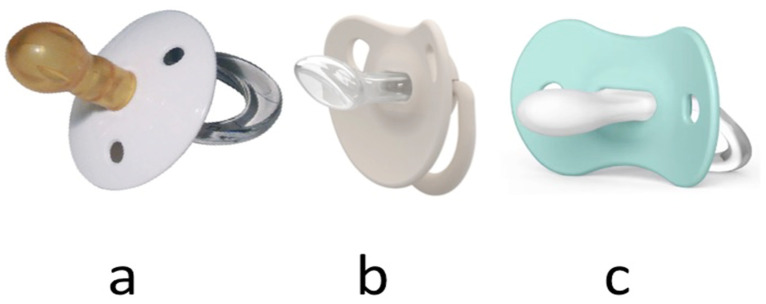
Different shapes of the pacifier. (**a**) Conventional, (**b**) anatomical, and (**c**) physiological.

It must be made of a single piece, have a large base in order to prevent the baby from inserting the pacifier completely into the oral cavity, and have a ring to be able to pull it. The nipple can be made of silicone or latex, although the latter material has recently been discouraged. As a result of the dental alterations it can cause, several designs have been described, differing in the shape and size of the teat (Figure 1):

- Conventional (cherry) (a): the shape of the nipple is round and large, and is not recommended due to its degree of interference with breastfeeding and the development of the palate.
- Anatomical (drop) (b): Its nipple has a flat shape and is wider at the top, similar to an inverted drop. This design is based on the idea that the pacifier should not interfere with the development of the baby’s mouth and teeth. Drop pacifiers are considered to reduce the pressure exerted on the palate and teeth, preventing possible malformations. However, it is not supported by many studies.
- Physiological (c): Although it is said to have a shape similar to that of the mother’s nipple during breastfeeding, the nipple of the pacifier is round and slightly elongated, with a flat base and a thin neck.

Regarding the size of the pacifier, it is recommended that it always be as small as possible and with the connection between the base and the nipple as thin as possible, because of its reduced interaction with the bite.

Due to the existence of different pacifier designs, the main objective of this work is to determine whether there is a lower prevalence of dental alterations with the use of a physiological pacifier in children with primary dentition.

## 2. Materials and Methods

### 2.1. Database Sources and Search Strategies

This scoping review was carried out following the Guidelines for Preferred Reporting Items for Systematic Reviews and Meta-Analyses (PRISMA) (2020) and was recorded in the International Prospective Register of Systematic Reviews (PROSPERO database) under protocol ID528195.

A search covering the period from 2010 to 2024 was conducted using PubMed, Embase, and Scopus. A selection of “MeSH terms” was established: “Malocclusion/etiology*”, “Pacifiers/adverse effects*”, and “Sucking Behavior*”. Then, using the Boolean commands {AND, OR}, search strategies could be applied to PubMed. An appropriate search strategy with identical keywords was also developed for Embase and Scopus.

The question of this review was: does the use of the physiological pacifier cause fewer dentomaxillary alterations than the other designs?

This question was formulated using the PICO format [10], including:

- Population: preschool children who had pacifier as a habit of sucking.
- Intervention: the use of pacifiers.
- Comparators: preschool children who do not use pacifiers.
- Outcome: Dental alterations in the vertical, horizontal, and sagittal plane.

### 2.2. Article Eligibility Criteria

This review included clinical trials conducted in human subjects (children), comparing different types of pacifier, and evaluating clinical and radiographic alterations at the orofacial level. Articles published more than 15 years ago, and studies without a description of the pacifier shape, with fewer than 50 participants, in vitro, written in a language other than English, case reports and case series, theses, reviews, systematic reviews, and meta-analyses were excluded.

### 2.3. Data Collection

The following elements were collected and considered relevant: study details (author, year, study design, country, number of participants, age of participants, sex, control group, and follow-up period), orofacial examination, type of pacifier used, maxillar alterations seen, and citations.

### 2.4. Evaluating the Risk of Bias

A risk of bias assessment was performed using the risk of bias in nonrandomised interventions (ROBINS-I) tool. It includes the risk of bias due to confounder factors (initiation and end of habit, initial malocclusion, presence of digital or object sucking), selection of participants for the study, classification of interventions, deviations from intended intervention, missing data, measurement of outcomes, and selection of reported results [11]. Two reviewers (I.R. and C.C.) independently ranked each study included and resolved any disagreement by reciprocal consultation.

## 3. Results

### 3.1. Study Selection

**Figure 2:**
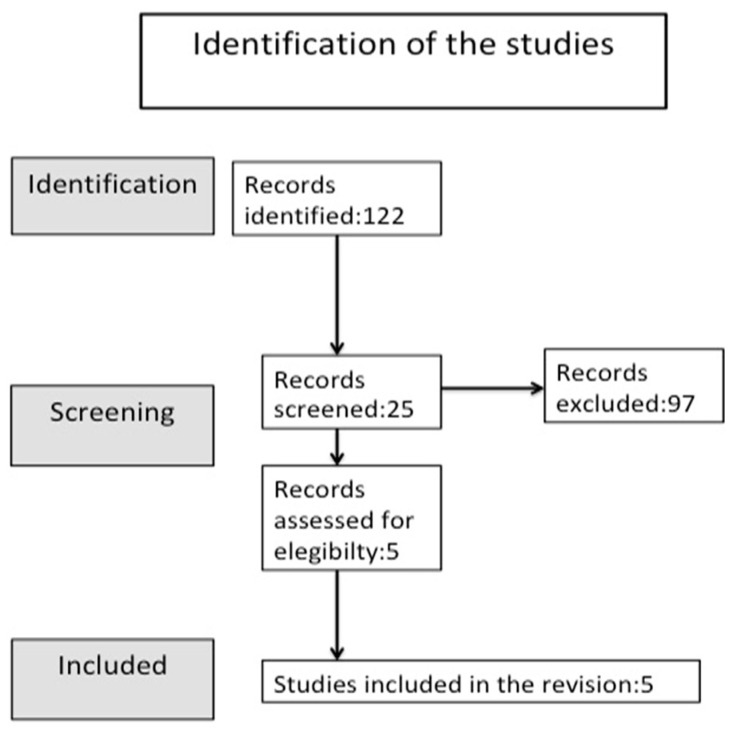
PRISMA flow chart for the selection and screening method for relevant publications.

A total of 122 articles were found in the initial search. The three reviewers independently reviewed the titles, abstracts, and full texts. The title of each piece of research retained was selected, and 97 references were excluded. Of the 25 articles, 5 were retained after reading the abstracts. A manual search was performed, and no additional eligible articles were found. After evaluating the full texts, five articles were considered eligible for this scoping review [12,13,14,15,16] (Figure 2).

### 3.2. Study Characteristics

The characteristics of the included studies are described in Table 1.

The first study was published in 2011 by Zimmer et al., and the latest was published in 2019 by Caruso et al. A total of three studies were conducted in Germany [12,15,16], one in Brazil [13], and one in Italy [14]. All included studies were observational studies, except for a randomized controlled trial [12]. The number of participants ranged from 63 [12] to 220 children [14]. The age of the participants ranged between 15 months [15,16] and 60 months [13]. The follow-up period was at least 12 months in all included studies, reaching three years in Caruso et al. [13] and Lima et al. [14]. The occlusal alterations examined were mainly: anterior open bite, posterior crossbite, overjet, and overbite.

### 3.3. Maxilar and Dental Alterations

In all of them, in general, the use of the pacifier caused malocclusions such as an anterior open bite, a posterior crossbite, an increased overjet, and an involvement of the overbite. The only study that examined a single type of pacifier was by Caruso et al. in 2019 [13], and the physiological pacifier was analyzed. The regression revealed a significant contribution of the initial sucking of the physiological pacifier to the prevalence of finger/thumbsucking, because children who started using the orthodontic pacifier very early, between 0 and 3 months, were less likely to develop fingersucking/thumbsucking compared to children who started after 3 months (OR 0.13, 95% CI 0.04 to 0.47, *p* = 0.0004). Another study carried out in 2017 was by Lima et al. [14], in which the physiological pacifier was compared with the conventional one. In the prevalence of open anterior bite, there was a significant difference between the use of conventional pacifiers compared to the use of physiological pacifiers (*p* = 0.027). Furthermore, only the use of conventional pacifiers resulted in a higher prevalence of posterior crossbite compared to the control group (*p* = 0.040). The physiological pacifier was also compared with the anatomical one in the studies by Zimmer et al. in 2011 [15] and 2016 [16]. Among the results of the study carried out in 2011, it was highlighted that 38% of the children who used the NUK pacifier (anatomical type) showed an open anterior bite, and 5% of the children who used the Dentistar pacifier (physiological type) did. Therefore, the incidence of open bites was significantly lower in the group of children who used the Dentistar pacifier (*p* < 0.001). In the study carried out in 2016, the results highlighted that 6.7% of the children who used the Dentistar pacifier had an anterior open bite and for the group of children who used the NUK pacifier, it was a percentage of 50.00%. Children with a statistically significant difference (*p* < 0.001). One of the five studies, that of Wagner and Heinrich-Weltzien [12] from 2016, was the use of three types of pacifier in two groups: anatomical and conventional and, on the other hand, physiological. The difference between the group of children who used the physiological pacifier and the group that used anatomical or conventional pacifiers was statistically significant for the increase in overjet (3.1 ± 0.2 mm vs. 3.6 ± 0.3 mm, respectively; *p* < 0.001) and anterior open bite (−1.2 ± 0.3 mm vs. −2.2 ± 0.3 mm; *p* < 0.001). Data from the specific studies are reported in Table 1.

### 3.4. Risk of Bias

The risk of bias judgements in ROBINS-I are depicted in Table 2. Four studies [13,14,15,16] were evaluated as having an overall moderate risk of bias, and one study [12] had a low risk of bias.

## 4. Discussion

The habit of sucking pacifiers has a skeletal effect, which will promote the establishment of maloclussion, because the stage of life in which it occurs coincides with growth and craniofacial development. The literature shows that this fact is due to the influence of the pacifier on the movement of the facial muscles, which are involved in the development of the jaws, and on the position of the tongue during habit, which will be low, not placed under the palate, leading to a tall and narrow one, and consequently to the corresponding maloclussion [17].

Regarding the prevalence of malocclusions among children who use pacifiers, it is approximately 38 to 94%. The appearance of an open anterior bite is between 17 and 96% of children using pacifiers, while the posterior crossbite is between 27 and 88%, with overjet in 52% of them [17]. In his meta-analysis, Poyak [18] demonstrated that the most notable changes are the increase in the prevalence of open anterior bite, posterior crossbite, narrowing of the intercuspal distances of the maxillary arch, and the broad and narrow palate.

In this work, a review of five articles has been carried out that have investigated the influence of the shape of the pacifier on alterations in occlusion. Very few contemporary studies compare occlusion in children using different forms of pacifier. When comparing the maloclussion of children who suck a pacifier, the physiological pacifier is the one with which the least alterations have been observed in this work. Some authors [13,14,19,20] refer to the physiological pacifier with the term “orthodontic”—in this review we have referred to it by the term “physiological”, considering that the “orthodontic” can cause confusion, since its function is not really to correct a malocclusion. We consider that there are no good pacifiers, but rather less bad pacifiers since all of them, when used for a long time, have the ability to modify the child’s bite.

Furthermore, studies also show that the frequency and intensity with which this habit is performed will increase the probability of developing maloclussion. Similarly, in the current systematic review by Schmid et al. [5], the physiological pacifier was shown to cause significantly fewer open bites than the conventional pacifier. In the systematic review by Medeiros et al. [19], only three studies were included after the selection process. Greater anterior open bite and overjet were identified with a conventional pacifier than with a physiological pacifier.

With respect to the physiological pacifier, it is characterized by having a narrower, flattened, and thinner suction zone. This flatter teat design attempts to mimic the shape of the mother’s nipple. The upper part is slightly rounded, and the lower part is flat. This more horizontal and elongated axis reduces pressure on the teeth, gums, and jaw. In addition, it allows the tongue to exercise more natural movement during sucking. This harmoniously accompanies the emergence of teeth and the growth of the jaws, and prevents excessive pressure on these structures, which could cause bite problems in the future. However, the sucking area of the conventional pacifier is larger and round, either all around or at the tip. Regarding the anatomical aspect, which is that it has a round and slightly elongated nipple, despite being said to be similar in shape to the mother’s nipple during breastfeeding, this is not true.

The first included studies were carried out by Zimmer et al. [15] and later in 2016 [16], both with a moderate risk of bias. In these two studies, the physiological pacifier was compared with the anatomical pacifier. The incidence of open anterior bite was significantly lower in the physiological pacifier group compared to the anatomical pacifier group (*p* < 0.001). The first study in the literature that showed the advantages of using a physiological pacifier over an anatomical pacifier or a conventional pacifier with respect to overjet and overbite was that of Wagner et al. [12], which has been included in the present systematic review due to its high quality. It should be noted that, although the differences observed are small, they are statistically significant. A more recent study of the present review was carried out by Lima et al. [14] in Brazil. This study, with a moderate risk of bias, compared the physiological pacifier with the conventional pacifier. No significant differences were found in the prevalence rates of malocclusion between the physiological pacifier group and the conventional pacifier group, except in the anterior open bite, which was more frequent in the conventional pacifier. However, adjusted logistic regression analyzes detected stronger associations between maloclussions and conventional pacifier use compared to a physiological pacifier. After adjustment, the posterior crossbites were only associated with the use of conventional pacifiers (adjusted OR = 10.7; 95% CI = 1.1–102.1). The most recent study in this review also compared a physiological pacifier with a conventional one, and was carried out by Caruso et al. [13], classifying it as having a moderate risk of bias. Logistic regression did not show any correlation between physiological pacifier suction and the presence of maloclussions.

However, in previous studies, there were differences in some results with this review, as well as controversial results. In the study by Adair et al. [17], although the prevalence rates of posterior crossbite and overbite did not differ between the conventional pacifier or physiological pacifier groups, the prevalence rates of anterior open bite and overjet did. In their analysis of 79 children aged 24–59 months, anterior open bite was higher in users of conventional pacifiers, corroborating the results described in this review. However, the excess jet was higher among those using a physiological pacifier compared to the conventional pacifier. In another study by Adair et al. [21], 82 children who had used a physiological pacifier were compared with 38 children who used a conventional pacifier. The results did not indicate differences in the prevalence rates of anterior open bite or overjet. Similar results were reported by de Zardetto et al. [22] who concluded that the prevalence rates of alterations in dental relationships and myofacial structures were higher in those who sucked pacifiers, conventional or physiological, compared to those who did not use them. In turn, Mesomo et al. [20] reported a high prevalence of posterior crossbite among physiological pacifier users compared to controls. The use of a conventional pacifier and physiological pacifier was a risk factor for anterior open bite, overjet, and tendency to Class II. These divergences could perhaps be partially explained by methodological differences between the studies. As a general rule, samples from previous studies comparing the use of a conventional pacifier and physiological pacifier were very small and did not adjust associations for potential confounders. Furthermore, some of the studies included children who had already stopped using pacifiers, which could have introduced bias into the evaluations. In turn, the studies in this systematic review used a larger population sample and some strategies were applied to control confounders, two of them having low risk of bias and the other three having moderate risk.

Most of the previous literature states that, with a long duration and high frequency of pacifier usage, there is a tendency to maloclussions for more than two years [23], or more than one year [24]. However, in the moderate-quality study by Caruso et al. [13], it was observed that the duration of physiological pacifier use was not associated with the prevalence of malocclusions, even when used for more than two years. In older children, the harmful effects that can be caused are clear. The effect of physiological pacifiers is often temporary, and only if its use extends to a mixed dentition period can it potentially create a narrow upper arch and maybe an increased overjet. Similar effects (a narrow upper arch, an increased overjet, proclined upper incisors, and retroclined lower incisor) can be created in a growing adolescent with a habit of finger-sucking/thumb-sucking [24].

This same study of Caruso et al. [13] obtained very interesting findings in that the use of physiological pacifiers does not promote the occurrence of poor oral habits in children. Only a few studies in the literature had analysed the relationship between pacifier sucking and other poor oral habits. In terms of interposition of the tongue between dental arches at rest, it was previously investigated in a sample of 36 preschool children using conventional pacifiers and was observed in 38.9% of children [23]. Despite what has been reported about conventional pacifiers, it could be hypothesised that children could experience improved safety and satisfaction due to unrestricted sucking, as previously reported for breastfeeding [25], so no other sucking actions are needed, leading to a low frequency of fingersucking/thumbsucking and other poor oral habits in children using physiological pacifiers.

It should be noted that there are very few studies that analyze the consequences of pacifier shape, so it has only been possible to discuss the results of the studies included in this systematic review with few studies in the literature.

Furthermore, in some of these investigations, the difficulty in following up on children has been highlighted, losing part of the sample. It is also not easy to obtain reliable data from parents, as duration data are often based on information provided by parents and therefore the uniformity of data collection could be affected.

Other limitations found were inadequate study designs, lack of examination of the duration and frequency of pacifier sucking, which plays an important role in the development of malocclusions, and lack of adequate follow-up with some malocclusions caused by the pacifier not being diagnosed. It will also be interesting to consider other variables that would influence the results, such as the children’s behavior.

The risk of bias in the included studies was mostly moderate. More studies with high-quality control groups and prospective approaches will be necessary.

Finally, intraoral measurements in children under 3 years of age due to their stage of development mean that co-operation is limited, and the measurements could have been more precise if impressions and photographs had been available.

Based on the findings of this systematic review, some recommendations are proposed:

- It would be appropriate to consult the pediatrician, pharmacist, and pediatric dentist about the best alternative to a pacifier and create programs that can inform parents about these types of everyday problem that are not given the importance they deserve. There is a wide variety of pacifiers in terms of material, shape, size, and decoration. This last aspect is one that should never be decided, since other variables that may affect the baby’s development must be taken into account. However, in recent times, cherry pacifiers (conventional ones) have become fashionable, which have been seen to lead to a more likely development of malocclusion in the child.
- More research is needed in this field, carrying out randomized controlled trials with larger samples, adequate follow-ups, and tools that control confounding factors (quantifying the frequency, intensity, duration of pacifier use, and the presence of other habits), to be able to continue reaching solid conclusions and be able to establish and recommend certain pacifiers to the children’s population from scientific societies. Studies in this review and those existing in the literature show a moderate risk of bias, which means that the findings should be evaluated with some care.
- Radical behaviour against its use should not be indicated. Despite controversies, the use of pacifiers can bring benefits to a child. Therefore, it is important to inform parents about its rational use, in order to avoid malocclusion and bring the expected benefits. Although frequency and duration are decisive in preventing occlusion, the indication of choosing an orthodontic pacifier could be suggested. More high-quality research is being carried out to support the recommendation of a physiological pacifier as a preventive measure of malocclusion, although it should be noted that most of the literature finds minor alterations with them and two studies with a low risk of bias have been presented in this review.

## 5. Conclusions

Taking into account the present evidence, physiological pacifiers reduce the occurrence of anterior open bite and overjet. More high-quality investigations with randomised controlled trials are required to support the physiological pacifier as a recommendation to prevent the development of the malocclusion trait.

## Figures and Tables

**Table 1:** Description of the included studies.

Author, Year, Country	Study Design	Sample Size	Age Range	Gender	CG	Follow-Up	Clinic Examination	Maxillodental Alterations	Citations
Caruso et al., 2019 [13], Italy	Cohort retrospective	198 c	36–60 months	96 male, 102 female	no	1–3 years old	MO, PC, interposition of tongue at rest, immature deglution, alteration in speech	PP does not favour the development of bad oral habits even if used for 2 years old and does not seem to correlate with MO unlike what is indicated in the literature on the CP.	20
Lima et al., 2017 [17], Brazil	Cohort prospective	220 c	24–36 months	not mentioned	yes	2–3 years old	AOB, OJ, PC, OB, molar and canine Angle’s Class	The prevalence of MO was higher in c with P. CP was associated with severe AOB and OJ.	16
Wagner and Heinrich-Weltzien, 2016 [12], Germany	RCT	63 c	16–24 months	not mentioned	yes	1 years old	AOB, OJ	PP had better measurements of OJ and OB than CP and AP.	5
Zimmer et al., 2016 [16], Germany	Cohort prospective	121 c	15–17 months	55 male, 66 female	yes	2 years old	AOB, OJ	The use of P may favor AOB. PP would cause less OB than AP	8
Zimmer et al., 2011 [15], Germany	Cohort prospective	121 c	15–17 months	57 male, 64 female	yes	1 year old	AOB, OJ, OB, molar and canine Angle’s Class	PP showed significantly less AOB than AP	3

RCT: randomized controlled trial; c: children; CG: control group; P: pacifier; PP: physiological pacifier; CP: conventional pacifier; AP: anatomical pacifier; MO: maloclussion; PC: posterior crossbite; OJ: overjet; OB; overbite; AOB: open anterior bite.

**Table 2:** Risk of bias evaluation according to ROBINS-I.

	Confounding	Selection of Participants	Classification of Interventions	Deviations from Intended Interventions	Missing Data	Measurement of Outcomes	Selection of Reported Results	Overall
Caruso et al. [13]	High	Moderate	Moderate	Moderate	Low	Moderate	Low	Moderate
Lima et al. [17]	Moderate	Moderate	Low	Moderate	Moderate	Low	Moderate	Moderate
Wagner et al. [12]	Low	Low	Low	Low	Moderate	Low	Moderate	Low
Zimmer et al. [16]	Moderate	Moderate	Low	Moderate	Moderate	Low	Low	Moderate
Zimmer et al. [15]	Severe	Moderate	Low	Moderate	Moderate	Low	Low	Moderate

## Data Availability

Data are contained within the article.
